# Overexpression of microRNA-99a attenuates heart remodelling and improves cardiac performance after myocardial infarction

**DOI:** 10.1111/jcmm.12242

**Published:** 2014-03-13

**Authors:** Qiaoling Li, Jun Xie, Ruotian Li, Jian Shi, Jiayin Sun, Rong Gu, Liang Ding, Lian Wang, Biao Xu

**Affiliations:** a Department of Cardiology, Drum Tower Clinical Medical Hospital, Nanjing Medical UniversityNanjing, China; b Department of Cardiology, Drum Tower Hospital, Nanjing University Medical SchoolNanjing, China

**Keywords:** microRNA, ischaemic heart disease, apoptosis, autophagy, gene therapy

## Abstract

MicroRNAs are involved in the regulation of various cellular processes, including cell apoptosis and autophagy. Expression of microRNA-99a (miR-99a) is reduced in apoptotic neonatal mice ventricular myocytes (NMVMs) subjected to hypoxia. We hypothesize that miR-99a might restore cardiac function after myocardial infarction (MI) by up-regulation of myocyte autophagy and apoptosis. We observed down-regulated miR-99a expression in NMVMs exposed to hypoxia using TaqMan quantitative reverse transcriptase-polymerase chain reaction analysis (RT-PCR). We also observed that miR-99a overexpression decreased hypoxia-mediated apoptosis in cultured NMVMs. To investigate whether overexpression of miR-99a *in vivo* could improve cardiac function in ischaemic heart, adult C57/BL6 mice undergoing MI were randomized into two groups and were intra-myocardially injected with lenti-99a-green fluorescent protein (GFP) or lenti-GFP (control). Four weeks after MI, lenti-99a-GFP group showed significant improvement in both left ventricular (LV) function and survival ratio, as compared to the lenti-GFP group. Histological analysis, western blotting analysis and electron microscopy revealed decreased cellular apoptosis and increased autophagy in cardiomyocytes of lenti-99a-GFP group. Furthermore, western blotting analysis showed inhibited mammalian target of rapamycin (mTOR) expression in the border zones of hearts in miR-99a-treated group. Our results demonstrate that miR-99a overexpression improves both cardiac function and survival ratio in a murine model of MI by preventing cell apoptosis and increasing autophagy *via* an mTOR/P70/S6K signalling pathway. These findings suggest that miR-99a plays a cardioprotective role in post-infarction LV remodelling and increased expression of miR-99a may have a therapeutic potential in ischaemic heart disease.

## Introduction

Ischaemic heart disease is the leading cause of diseases with high morbidity and mortality worldwide [1,2]. It is known that prolonged ischaemia of myocardium results in an irreversible loss of cardiac myocytes (necrosis and apoptosis) [3,4], leading to pathological myocardial remodelling [5]. Thus, how to attenuate the loss of myocytes in the adverse pathological remodelling has long been focused on as an efficient strategy to improve the post-infarct prognosis.

MicroRNAs are 21 to 23 nt non-coding small RNAs that act primarily as negative regulators of the post-transcriptional gene expression. There is emerging evidence for the cardioprotective role of microRNAs in ischaemic heart. MiR-21 and miR-24 were proven to protect against myocytes apoptosis under ischaemia reperfusion [6,7]. MiR-210 and miR-1 were proven to improve cardiac function after myocardial infarction (MI) by enhancing angiogenesis and inhibiting cardiomyocyte apoptosis [8,9]. Human miR-99a is encoded by 21st chromosomes and its targets include a variety of molecules involved in cardiovascular pathology, such as mammalian target of rapamycin (mTOR), SWItch/Sucrose NonFermentable (SWI/SNF)-related matrix-associated actin-dependent regulator of chromatin subfamily A member 5 (SMARCA5), SWI/SNF-related matrix-associated actin-dependent regulator of chromatin subfamily D member 1 (SMARCD1) and fibroblast growth factor receptor 3 (FGFR3) [10–13]. Published data show that the expression of miR-99a was decreased in apoptotic cardiomyocyte upon exposure to hypoxia *in vitro* [8], suggesting that miR-99a is associated with hypoxia-induced cardiomyocyte apoptosis. Eulalio *et al*. identified that miR-99a mimics could significantly increase neonatal cardiomyocytes proliferation [14]. However, studies on the role of miR-99a in MI and subsequent cardiac remodelling are very limited. The precise role and therapeutic potential of miR-99a in MI is still unknown.

It has been suggested that miR-99a protected heart against infarction by regulation of cardiomyocyte survival. To test this concept, we overexpressed miR-99a in the ischaemic heart by lentiviral delivery of miR-99a. We observed improved heart function and attenuated pathological myocardial remodelling in MI mice treated with miR-99a. Mechanistically, the protective role of miR-99a in MI was largely because of its inhibition of apoptosis and promotion autophagy.

## Materials and methods

Animal care and sacrifice, primary culture of neonatal mice ventricular myocytes (NMVMs), cell infection and hypoxic conditions, *in vivo* study design, ultrasonic cardiogram (UCG), microRNA and mRNA quantification, western blotting analysis and statistical analysis are described in the ‘Materials and methods’ section in Data S1.

## Results

### Decreased miR-99a expression in infarcted hearts and hypoxic NMVMs

**Fig. 1:**
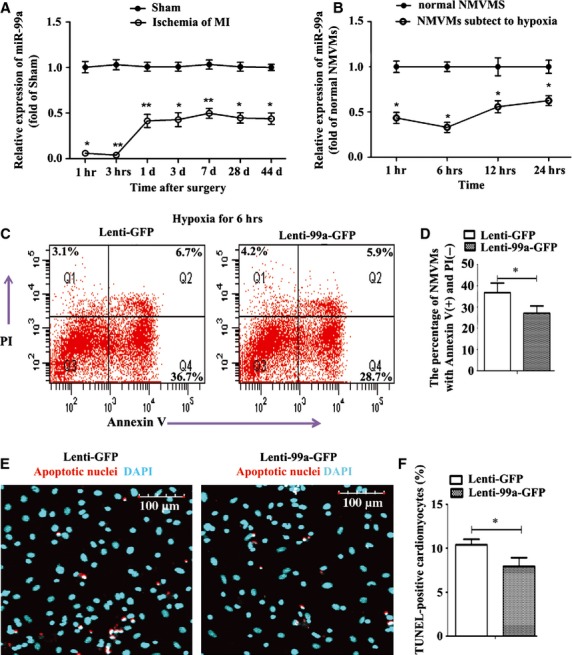
Expression profile of miR-99a in ischaemic heart and neonatal mice ventricular myocytes (NMVMs) under hypoxia. (**A**) MiR-99a expression in the border zone of infracted heart was assessed by RT-PCR at the indicated times after surgery (*n* = 5, each time-point per group). MiR-99a expression was normalized to the U6 expression and expressed as fold change relative to sham group. (**B**) NMVMs were exposed to 1% oxygen and miR-99a expression in NMVMs was analysed at different time-points. (**C** and **D**) MiR-99a overexpressing or control NMVMs were exposed to hypoxia for 6 hrs, and cell apoptosis was measured by FACS-Annexin V/propidium iodide (PI) staining. Annexin V (+) PI (−) cells were considered to be early apoptotic cells. (**E** and **F**) NMVMs were subjected to hypoxia for 6 hrs. Apoptotic nuclei were detected using TUNEL staining (red) and with 4′-6-diamidino-2-phenylindole (DAPI) nuclear counterstaining (cyan; **E**). Quantification of apoptosis following TUNEL staining (**F**). Values expressed as mean ± SEM from three independent experiments; *P < 0.05, **P < 0.01.

The expression profile of miR-99a was markedly different between MI group and control (sham) group throughout the 44-day observation period. MiR-99a expression dramatically decreased up to 94% 1 hr after infarction, then recovered to only 41 ± 7% of the miR-99a level in the control group 24 hrs after infarction and maintained thereafter 43–50% of the miR-99a levels in the control group over the 44-day period (Fig. 1A). To confirm our observation that miR-99a expression decreased in infarcted heart, we assessed miR-99a expression in NMVMs under hypoxia. The purity of cultured myocytes was estimated ≥95% using the cardiac-specific marker α-sarcomeric actin staining (Figure S1A). We observed a progressive decrease in miR-99a expression in NMVMs ranging from 43% at 1 hr of hypoxia to 21% at 6 hrs of hypoxia. MiR-99a levels gradually returned to ∼50% of normoxia levels at 24 hrs of hypoxia (Fig. 1B). The significantly decreased miR-99a expression in both infracted hearts and NMVMs under hypoxia suggests an important role for miR-99a in ischaemic injury.

Previous study had shown the regulation of miR-99a expression by MEK1/2/MAPK pathway [13]. To investigate whether MEK1/2/MAPK pathway is involved in the suppressed miR-99a expression that we observed, we evaluated ERK1/2 and phosphorylated ERK1/2 levels in hypoxic NMVMs. Western blotting analysis revealed that the phosphorylation of ERK1/2 was up-regulated within 1 hr of hypoxia and this up-regulation lasted for about 12 hrs of hypoxia (Figure S2A and B). We also assessed miR-99a expression in hypoxic NMVMs in the presence of a MEK1/2 inhibitor (U0126). As shown in Figure S2B, there was a fourfold increase in miR-99a expression in U0126-treated NMVMs after 6 hrs of hypoxia and miR-99a expression maintained at a high level (approximately twofold higher than control, Figure S2C) even after 24 hrs of hypoxia. These data suggest that the down-regulating effect of hypoxia on miR-99a expression in NMVMs is mediated by the MEK1/2/MAPK signalling pathway.

### Anti-apoptotic function of miR-99a in NMVMs

Cardiomyocyte apoptosis contributes to post-infarction cardiac remodelling and subsequent cardiac dysfunction [5]. To identify whether miR-99a overexpression protects NMVMs against apoptosis under hypoxic stress, we infected NMVMs with lenti-99a-green fluorescent protein (GFP) or lenti-GFP (Figure S1B) and assessed miR-99a expression using RT-PCR analysis. MiR-99a expression was about 30-fold higher in the lenti-99a-GFP group than the lenti-GFP group (Figure S1C). Early and late apoptosis of NMVMs under hypoxia stress were assessed by flow cytometry using annexin V/propidium iodide (PI) double staining and TUNEL assay respectively. As shown in Figure 1C and D, the percentage of annexin V-positive apoptotic cells was significantly decreased in miR-99a-overexpressed myocytes 6 hrs after hypoxia compared to the lenti-GFP group, indicating that early apoptosis of hypoxic myocytes was reduced by miR-99a overexpression. The percentage of TUNEL-positive myocytes was also lower in the lenti-99a-GFP-treated group than the lenti-GFP-treated group (Fig. 1E and F). Taken together, these data demonstrate that miR-99a attenuates hypoxia induced early and late apoptosis in myocytes.

### Lentiviral vectors expression in myocardium

**Fig. 2:**
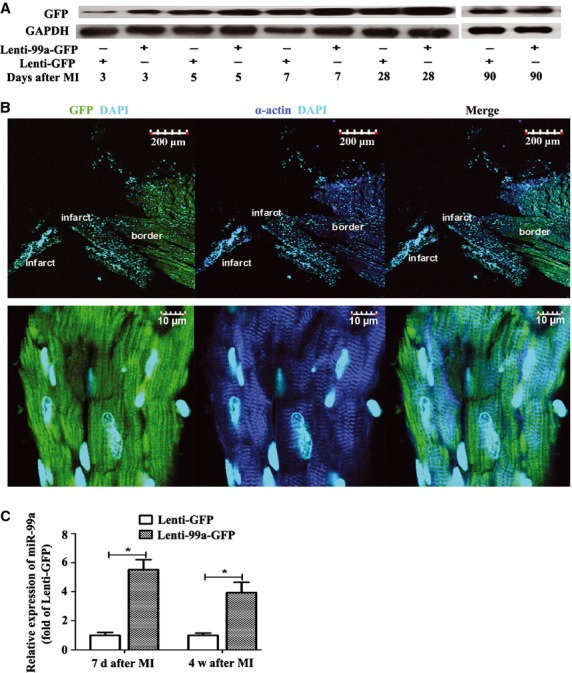
Expression of green fluorescent protein (GFP) and miR-99a after lentiviral delivery. (**A**) Expression of GFP in the border zone of infarct heart after lentivirus delivery was assessed by western blotting analysis at indicated times after myocardial infarction (MI; *n* = 5 each time-point per group). (**B**) Immunofluorescence staining of GFP (green) in the border zone around the injection sites indicates successful expression of exogenous miR-99a (upper pannel). GFP expression (green) is mainly observed in cardiomyocytes defined as α-sarcomeric actin positive cells (blue; lower pannel). DAPI nuclear staining in cyan. (**C**) MiR-99a expression in the border zone of infarct heart was assessed by TaqMan RT-PCR at 7 days and 28 days after MI (*n* = 5 each group). Values expressed as mean ± SEM; *P < 0.05.

Expression of lentiviral vectors in myocardium was evaluated by western blotting analysis and immunofluorescence analysis using GFP as a molecular marker. Western blotting analysis showed that GFP was detectable 3 days after lentivirus delivery and was still present at 90 days after lentivirus transduction (Fig. 2A). Immunofluorescence staining of GFP combined with α-sarcomeric actin was performed on heart slices 28 days after lentivirus delivery to assess the morphological distribution of exogenous gene expression (Figure S3A). Green fluorescent protein-positive cells were mainly located in the border zone (Fig. 2B, upper panel) despite a sporadic distribution in the remote zone (date not shown). High-magnification views showed that the majority of the GFP-positive cells were myocytes, as demonstrated by double-positive staining of GFP and α-sarcomeric actin (Fig. 2B, lower panel). The expression of miR-99a in the border zone of heart after lentivirus delivery was evaluated using a TaqMan RT-PCR kit. As shown in Figure 2C, miR-99a expression was 5.52 ± 0.69-fold higher in miR-99a group 7 days after MI, and maintained 3.94 ± 0.71-fold higher 28 days after MI. To exclude the possibility that some lentiviral vectors may spill over to the circulation and increase miR-99a expression in other tissues, we tested miR-99a expression in aorta, livers, kidneys and lungs after lentiviral vector delivery into heart using a TaqMan RT-PCR kit or immunofluorescent staining of GFP. We barely found expression of miR-99a or GFP delivered by lenti-miR-99a vectors in aorta, liver, kidney and lung (data not shown). All these results indicated a successful *in vivo* gene delivery and expression in the border zone of ischaemic myocardium.

### Improvement in survival and cardiac function of MI mice by miR-99a overexpression

**Fig. 3:**
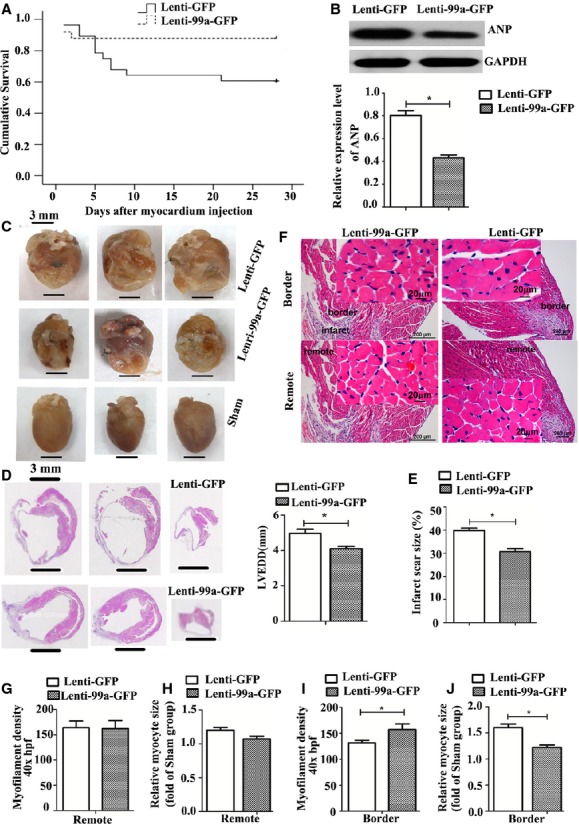
MiR-99a gene therapy improves mice survival, cardiac morphology and function after myocardial infarction (MI). (**A**) Kaplan–Meier survival curve of MI mice treated with lenti-99a-GFP (*n* = 25) or lenti-GFP (*n* = 28), χ^2^ = 4.22, *P* = 0.04. (**B**) Atrial natriuretic peptide expression in infracted hearts following lenti-99a-GFP delivery was assessed by western blotting analysis. Graph below shows densitometric quantification. (**C**) Hearts from lenti-GFP mice (top row) appear more spherical relative to lenti-99a-GFP (middle row) and sham mice (bottom row); bars = 3 mm. (**D**) Paraffin-embedded sections (base, mid region, and apex sequentially) of myocardium were stained with haematoxylin and eosin 4 weeks after lenti-99a-GFP (bottom row) or lenti-GFP (top row) delivery; bars = 3 mm. (**E**) Quantification of infarct scar size. (**F**) Representative image of myocytes in the border and remote zones of hearts from mice treated with lenti-99a-GFP or lenti-GFP; bars = 20 μm (high magnification), 200 μm (low magnification). (**G**–**J**) Analysis of myofilament density, and myocyte size in haematoxylin and eosin-stained sections (*n* = 5 per group). Values expressed as mean ± SEM; *P < 0.05.

During the first 3 days after lentiviral delivery in the border zone, lenti-99a-GFP group and lenti-GFP group showed similar mortality rates (Fig. 3A). However, the rates of overall survival of lenti-99a-GFP group were significantly higher than those of lenti-GFP group at 28 days after lentiviral delivery (Fig. 3A, 88% *versus* 60%). No deaths occurred in the sham group of mice.

Echocardiography (UCG) was performed to evaluate cardiac function of MI mice at 3 days, 1 week (Table 1) and 4 weeks (Table 2 and Figure S3B) after lentivirus delivery. Mice from both lenti-99a-GFP and lenti-GFP groups exhibited significantly impaired cardiac functional at 3 days after surgery. No noticeable difference was observed between the two groups at this time-point. However, the lenti-99a-GFP group demonstrated improved per cent ejection fraction (%EF), fractional shortening (%FS), left ventricular (LV) end-systolic diameter (LVESD) and end-diastolic diameter (LVEDD) in lenti-GFP group 1 week after lentivirus infection. And, this improvement was sustained over 4 weeks after lentivirus infection. In addition to UCG, hemodynamic analysis revealed a higher systolic blood pressure in lenti-99a-GFP group than the lenti-GFP group. The lenti-99a-GFP group also demonstrated higher diastolic blood pressure and lower heart rate compared to lenti-GFP group though no statistically significant difference was found. Furthermore, we observed no significant difference in cardiac function between lenti-99a-GFP-treated normal hearts and lenti-GFP-treated normal hearts (Table 1), suggesting that miR-99a overexpression does not affect cardiac function under non-surgical conditions.

**Table 1:** UCG was performed 3 days and 1 week after surgery

	Sham (*n* = 11)	Sham+Lenti-GFP (*n* = 5)	Lenti-GFP (*n* = 25)	Sham+Lenti-99a-GFP (*n* = 6)	Lenti-99a-GFP (*n* = 22)
Three days after surgery					
EF%	70.97 ± 1.01†	70.32 ± 3.52	57.46 ± 1.55	70.70 ± 1.96	58.71 ± 1.43*
FS%	39.12 ± 0.45†	38.94 ± 2.92	29.34 ± 1.28	39.20 ± 1.68	30.34 ± 1.45*
LVEDD (mm)	3.17 ± 0.13†	3.57 ± 0.18	3.34 ± 0.17	3.52 ± 0.15	3.56 ± 0.16*
LVESD (mm)	1.93 ± 0.18†	2.18 ± 0.11	2.36 ± 0.18	2.14 ± 0.18	2.48 ± 0.22*
One week after surgery					
EF%	75.25 ± 2.45†	73.34 ± 1.86	42.89 ± 3.11	74.78 ± 1.59	48.09 ± 2.97*
FS%	43.06 ± 2.19†	41.71 ± 1.55	20.83 ± 1.55	42.65 ± 1.41	23.77 ± 1.68*
LVEDD (mm)	3.46 ± 0.11‡	3.86 ± 0.08‡	4.08 ± 0.12	3.47 ± 0.05	3.87 ± 0.12*
LVESD (mm)	1.97 ± 0.13†	2.25 ± 0.10†	3.23 ± 0.16	1.99 ± 0.05	2.95 ± 0.15*

EF: Ejection fraction; LVEDD: LV end-diastolic diameter; LVESD: LV end-systolic diameter; %FS: percent fractional shortening; GFP: green fluorescent protein; UCG: ultrasonic cardiogram.

* *P* < 0.05, lenti-99a-GFP *versus* sham;

† *P* < 0.05, lenti-GFP *versus* sham;

‡ *P* < 0.05, Sham+Lenti-GFP *versus* Sham+Lenti-99a-GFP.

**Table 2:** UCG was performed 4 weeks after surgery

	Sham (*n* = 11)	lenti-GFP (*n* = 17)	lenti-99a-GFP (*n* = 22)
EF%	75.57 ± 0.69‡	39.61 ± 1.31†	48.11 ± 1.14*
FS%	43.35 ± 0.62‡	19.32 ± 0.71†	23.90 ± 0. 62*
Systolic pressure (mmHg)	118.79 ± 3.62‡	86.64 ± 4.54†	102.21 ± 3.11*
Diastolic pressure (mmHg)	83.12 ± 4.34‡	44.91 ± 4.11	57.93 ± 4.15*
Heart rate (beats/min)	428.41 ± 23.66	493.37 ± 31.61	439.36 ± 47.66
LVEDD (mm)	3.46 ± 0.10‡	4.97 ± 0.24†	4.10 ± 0.13*
LVESD (mm)	1.96 ± 0.06‡	4.01 ± 0.19†	3.12 ± 0.18*

* *P* < 0.05, lenti-99a-GFP *versus* sham;

† *P* < 0.05, lenti-99a-GFP *versus* lenti-GFP;

‡ *P* < 0.05 lenti-GFP *versus* sham.

It has been reported that atrial natriuretic peptide (ANP) levels closely correlate with the severity of LV dysfunction [15]. We assessed ANP expression in lenti-GFP and lenti-99a-GFP mice by Western blotting analysis. As shown in Figure 3B, ANP expression was significantly up-regulated in lenti-GFP group compared to lenti-99a-GFP group, suggesting the deterioration of LV function in lenti-GFP group. All these results indicate that overexpression of miR-99a improve heart function after MI.

### Assessment of ventricular morphology, myocyte size and myofilament density

Heart undergoes structural remodelling after infarction, resulting in a more spherical shape. Hearts from lenti-99a-GFP group showed less spherical shape than those from lenti-GFP group 4 weeks after MI, reflecting attenuated global cardiac remodelling in mice treated with miR-99a (Fig. 3C). Ventricular morphology was assessed using three blocks (base, mid region and apex) of each heart and averaged (Fig. 3D). Hearts from lenti-99a-GFP group showed reduced infarct size and LVEDD compared to lenti-GFP group (Fig. 3E), indicating that LV remodelling is improved by miR-99a overexpression.

Myocyte size and myofilament density were analysed in both the border and the remote zones (Fig. 3F). Increased myofilament density and decreased myocyte size were observed in the border zone of lenti-99a-GFP group compared to lenti-GFP group (Fig. 3I and J), while no significant difference in either myofilament density or myocyte size was observed in the remote zone between the two groups (Fig. 3G and H), suggesting that miR-99a overexpression attenuates myocyte hypertrophy and secondary cell loss in the border zone after MI.

Interstitial fibrosis is the hallmark of cardiac remodelling. As shown in Figure S3C, collagen volume fraction was reduced in the border zone of lenti-99a-GFP heart, suggesting suppressed interstitial fibrosis in heart treated miR-99a.

### Attenuation of apoptosis and regulation of autophagy by miR-99a overexpression *in vivo*

**Fig. 4:**
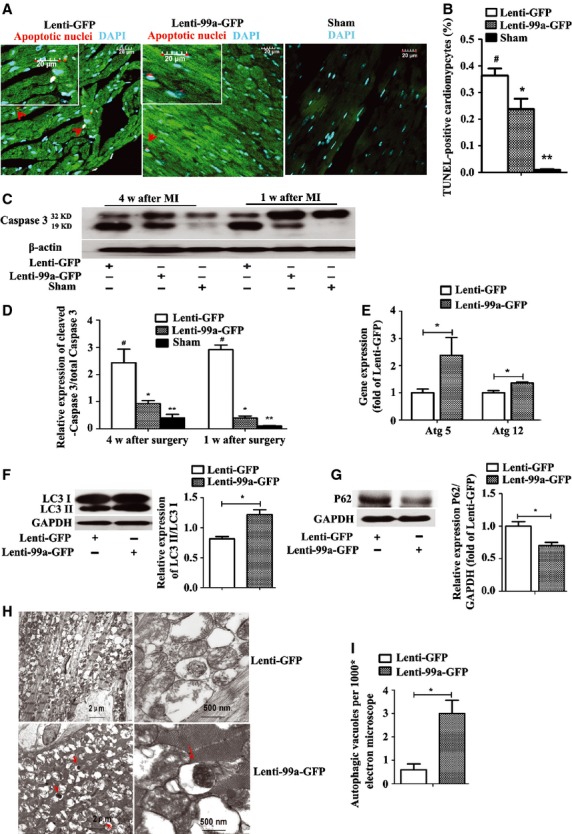
MiR-99a regulates autophagy and attenuates apoptosis after myocardial infarction (MI). (**A** and **B**) Apoptosis in lenti-99a-GFP or lenti-GFP infected myocytes (GFP+, green) and sham group was evaluated by TUNEL assay. TUNEL-positive myocytes (red) was significantly reduced in lenti-99a-GFP group compared to the lenti-GFP control group, whereas few TUNEL-positive cells were detectable in the sham group. DAPI nuclear staining in cyan. (**C** and **D**) The cleavage of caspase 3 at 19 kD was decreased in lenti-99a-GFP group. Graph below shows densitometric quantification (*n* = 5 per group). (**E**) mRNA expression levels of autophagic marker genes (Atg 5, Atg 12) in hearts treated with lenti-99a-GFP or lenti-GFP (*n* = 4 per group). (**F** and **G**) Four weeks after surgery, the expression of LC3II/LC3I (**F**) and P62 (**G**) in the border zone of infracted heart of lenti-99a-GFP group or lenti-GFP group was assessed by western blotting analysis (*n* = 5 per group). (**H** and **I**) Electron micrographs show more autophagic vacuole formation in myocyte of MI mice treated with miR-99a. Quantification of autophagy by measuring the average number of autophagic vacuoles per 10000* filed in electron microscope images; scale bars = 2 μm and 500 nm. Values expressed as mean ± SEM. **P < 0.05, lenti-99a-GFP *versus* sham; *P < 0.05, lenti-99a-GFP *versus* lenti-GFP; #*P* < 0.05 lenti-GFP *versus* sham.

Studies show that cell apoptosis and autophagy contribute to LV remodelling and dysfunction [16,17]. As microRNAs are involved in the regulation of cell apoptosis and autophagy, we investigated whether miR-99a overexpression attenuated LV remodelling by regulation of cell apoptosis and autophagy after MI. Significantly reduced apoptotic (TUNEL positive) cells were observed in the border zone of hearts from lenti-99a-GFP group compared to lenti-GFP group (Fig. 4A and B), suggesting the anti-apoptotic role for miR-99a. This observation was confirmed by assessment of the cleavage of caspase 3 by western blotting analysis, as decreased activation/cleavage of caspase 3 in the border zones of hearts after miR-99a treatment was observed (Fig. 4C and D).

Autophagy is a process that has recently been associated with myocyte survival in ischaemia. The ratio of LC3-II to LC3-I was used to assess autophagy in heart tissue and the autophagy substrate p62 level was associated with autophagic flux [18,19]. We observed up-regulation of several autophagic molecular markers including Atg5 and Atg 12 in lenti-99a-GFP group compared to lenti-GFP group (Fig. 4E). More importantly, the LC3 lipidation level (LC3II/LC3I ratios) was increased and the autophagy substrate p62 level was decreased in the border zone of miR-99a-treated hearts 4 weeks after MI (Fig. 4F and G), suggesting that miR-99a overexpression leads to an increased autophagic flux and enhanced autophagy in myocytes in response to myocardial ischaemia and infarction. The enhanced autophagy in miR-99a-treated heart was further confirmed by the increased autophagic vacuoles in miR-99a overexpressing cardiomyocytes as revealed by electron microscope (Fig. 4H and I).

### Regulation of mTOR/P70/S6K pathway by miR-99a

**Fig. 5:**
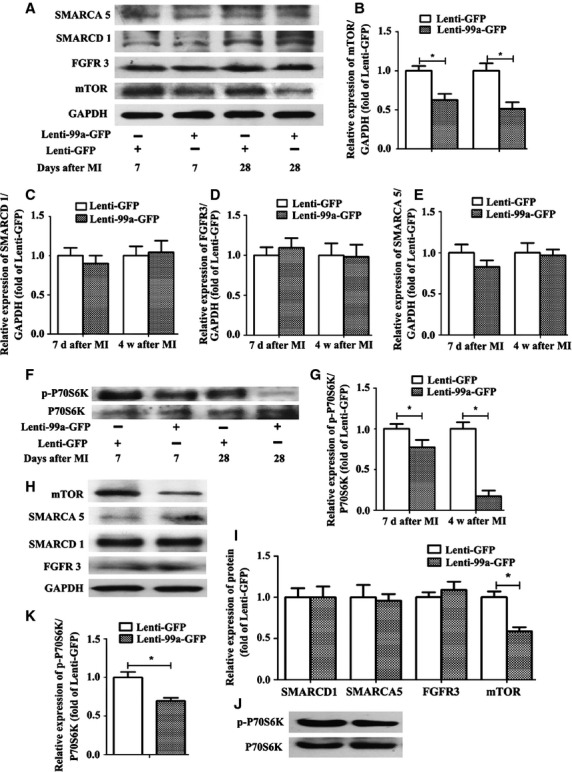
MiR-99a overexpression negatively regulates mTOR/p70/S6 kinase signalling pathway. (**A**–**E**) Protein samples for western blotting analysis were extracted from heart border zone tissues obtained at 7 days and 4 weeks after myocardial infarction (MI) *n* = 5 per group. (**A**) Expression of mTOR and other known miR-99a molecular targets including SMARCA5, SMARCD1 and FGFR3 in the border zone. (**B**–**E**) Graph shows densitometry quantification of the expression of mTOR, SMARCA5, SMARCD1 and FGFR3 in the border zone assessed by western blotting analysis. (**F** and **G**) Phosphorylation of p70/S6-kinase in the border zone of hearts of MI mice treated with lenti-GFP or lenti-99a-GFP. Graph shows densitometry quantification. (**H**–**K**) Protein samples for Western blotting analysis were extracted from neonatal mice ventricular myocytes (NMVMs) transfected with lenti-99a-GFP for 96 hrs. (**H** and **I**) Western blotting analysis shows expressions of mTOR, SMARCA5, SMARCD1 and FGFR3 in miR-99a overexpressing NMVMs. (**J** and **K**) Phosphorylation of p70/S6-kinase in miR-99a overexpressing NMVMs. Values expressed as mean ± SEM; *P < 0.05.

Data exist show that mTOR expression can be regulated by miR-99a, and mTOR/P70/S6K signalling pathway plays an important role in cardioprotection from ischaemia [10,13,17,20]. To investigate whether miR-99a overexpression improves heart function in MI mice by down-regulation of mTOR/P70/S6K pathway, we assessed mTOR and P70/S6K expression in miR-99a-lentivirus-infected heart and NMVMs. Western blotting analysis showed that both mTOR expression (Fig. 5A, B and H, I) and the activation modifications of P70/S6K (Fig. 5F, G and J, K) were decreased in miR-99a-overexpressing heart tissue and NMVMs. Interestingly, there was no significant decrease in mRNA transcriptional level of mTOR in miR-99a-overexpressing NMVMs (date not shown), suggesting that miR-99a regulates mTOR expression at a post-transcriptional level. To investigate whether other miR-99a targets besides mTOR are involved in the cardioprotective effect of miR-99a, we assessed the expression of other known miR-99a targets, including SMARCA5, SMARCD1 and FGFR3. We found no significant change in the expression of these molecules in either miR-99a-treated heart tissue (Fig. 5C–E) or miR-99a-treated NMVMs (Fig. 5H and I).

Taken together, all above suggest that miR-99a overexpression may protect heart against ischaemia injury *via* an mTOR/P70/S6K signalling pathway.

## Discussion

Cardiac remodelling is a determinant of the development of heart failure after MI. In response to ischaemic injury, progressive LV remodelling, including myocyte death, hypertrophy, fibrosis, occurs in both the ischaemic and the remote non-infarcted myocardium. Developing strategies to reduce post-MI cardiac remodelling is one of the major therapeutic challenges of modern cardiology. In the present study, we demonstrated a novel therapeutic strategy for the treatment of MI based on miR-99a overexpression. Lentivirus-mediated miR-99a delivery improved survival rate and cardiac function in mice after MI. And, the attenuated pathological LV remodelling attributes to enhanced autophagy and inhibited apoptosis of the border zone myocytes following MI.

Hu *et al*. [8] reported that the expression of miR-99a decreased in apoptotic myocytes subjected to hypoxia. In line with their finding, we observed that miR-99a was down-regulated in ischaemic myocardium and myocytes exposed to hypoxia. Previous study has shown the regulation of miR-99a expression in tumours by MEK1/2/MAPK pathway [13]. In this study, we observed the activation of ERK1/2 in myocyte under hypoxia. More importantly, the hypoxia-induced reduction in miR-99a expression is reversed by pharmacological inhibition of MEK1/2. Considering the previous finding that MEK1/2-ERK1/2 kinase in cardiomyocytes exerts rapid response to hypoxia stress [21], we think the decrease in miR-99a expression in the very early stage of hypoxia is because of the activation of MEK1/2-ERK1/2 kinase.

Cardiac myocyte apoptosis and autophagy play important roles in unfavourable LV remodelling after MI [5,22]. We found that miR-99a could prevent both cultured myocytes and myocardium against hypoxia-induced apoptosis. Therefore, decreased myocardial apoptosis is one of the mechanisms by which overexpressed miR-99a attenuates post-MI cardiac remodelling. Autophagy is a cell survival mechanism that involves degradation and recycling of cytoplasmic components, such as long-lived proteins and organelles [23]. Normally, autophagy is a homeostatic mechanism for maintaining myocyte size and global cardiac function. While in heart remodelling, it is an adaptive response that protects cells from hemodynamic stress [24]. Previous studies have demonstrated that autophagy protected myocytes against apoptosis in the chronically ischaemic swine myocardium [25,26]. In the present study, we found that miR-99a overexpression induced cell autophagy in the border myocardium, which might serve as another mechanism contributes to improve cardiac remodelling after miR-99a overexpression.

Mammalian target of rapamycin has been identified as the direct target of miR-99a by multiple studies using reporter gene assays [10,13,20]. Mammalian target of rapamycin is involved in pathological cardiac remodelling and heart failure. The role of mTOR in ischaemic heart disease is rather complicated. Genetic ablation of mTOR results in myocyte apoptosis and cardiac failure [27]. Cardiac mTOR overexpression protects against ischaemia-reperfusion (I/R) [28], while inhibition of mTOR have protective effects when the heart suffers from ageing, ischaemia and stress stimulus [29–33]. Recent studies show that mTORC1 is activated by pathological situations to induce pathological cardiac remodelling in heart [30–34]. Nevertheless, the multifaceted role of mTOR in heart may result from different models and stimulus applied in these studies. Our study demonstrates that mTOR is negatively regulated by miR-99a in both NMVMs and myocardium. As a key kinase, mTOR and its downstream signalling pathways are involved in cell death/survival and cell autophagy [35–37]. Inhibition of mTOR inhibition by everolimus, a derivative of rapamycin, protects the heart from ischaemic injury by promoting autophagy [19,38]. In this study, we observed that miR-99a overexpression effectively down-regulated the mTOR pathway, which may lead to the suppressed apoptosis and increased autophagy in injured myocytes. The mTOR pathway has been reported to play a pivotal role in the cross-talk between apoptosis and autophagy [39]. It is possible that down-regulated mTOR pathway promotes autophagy immediately after ischaemia to improve cell survival. With increasing stresses, the autophagic activity may be insufficient and mTOR pathways begin to protect cell from death by inhibiton of apoptosis. However, how mTOR signalling pathway precisely regulates switch between apoptosis and autophagy in miR-99a overexpressing myocytes needs further investigation.

Lentiviral vectors can transduce non-dividing cells and ensure stable and long-term transgene expression compared to adenoviral vectors [40]. Therefore, we used the lentiviral vectors to deliver and express miR-99a in myocytes in the present study. Besides established viral vector systems, chemically synthesized miRNA mimics are also useful tools for enhancing the expression of miRNAs. However, problems such as relatively low *in vivo* efficacy, difficulties in maintaining sufficient concentration of miRNA mimics in target tissue and potential side effects of miRNA mimics need to be resolved before they can be used for safe and effective miRNA therapy [41]. Nevertheless, it would be interesting to investigate the effect of the mir-99a by injection of a mir-99a mimic into the infarct heart in the future, as they can be present in the injected sites within the first 24 hrs post-MI.

In conclusion, our study demonstrates that overexpression of miR-99a improves post-MI cardiac function by up-regulating autophagy, inhibiting apoptosis and attenuating pathological remodelling. The regulation of mTOR signalling pathway by miR-99a may contribute to the cardioprotective role of miR-99a. Therefore, overexpression of miR-99a may serve as a new target for the treatment of ischaemic heart disease.

## Supporting information

**Figure S1** Purity and lentiviral infection of NMVMs.

**Figure S2** The activity of ERK1/2 was up-regulated under hypoxia.

**Figure S3** *In vivo* lentivirus expression.
