# Supplementary figures and images for: Overexpression of microRNA-99a attenuates heart remodelling and improves cardiac performance after myocardial infarction
Source: J Cell Mol Med. 2014 Mar 13;18(5):919–28. doi: 10.1111/jcmm.12242 (PMC4119397; doi:10.1111/jcmm.12242)

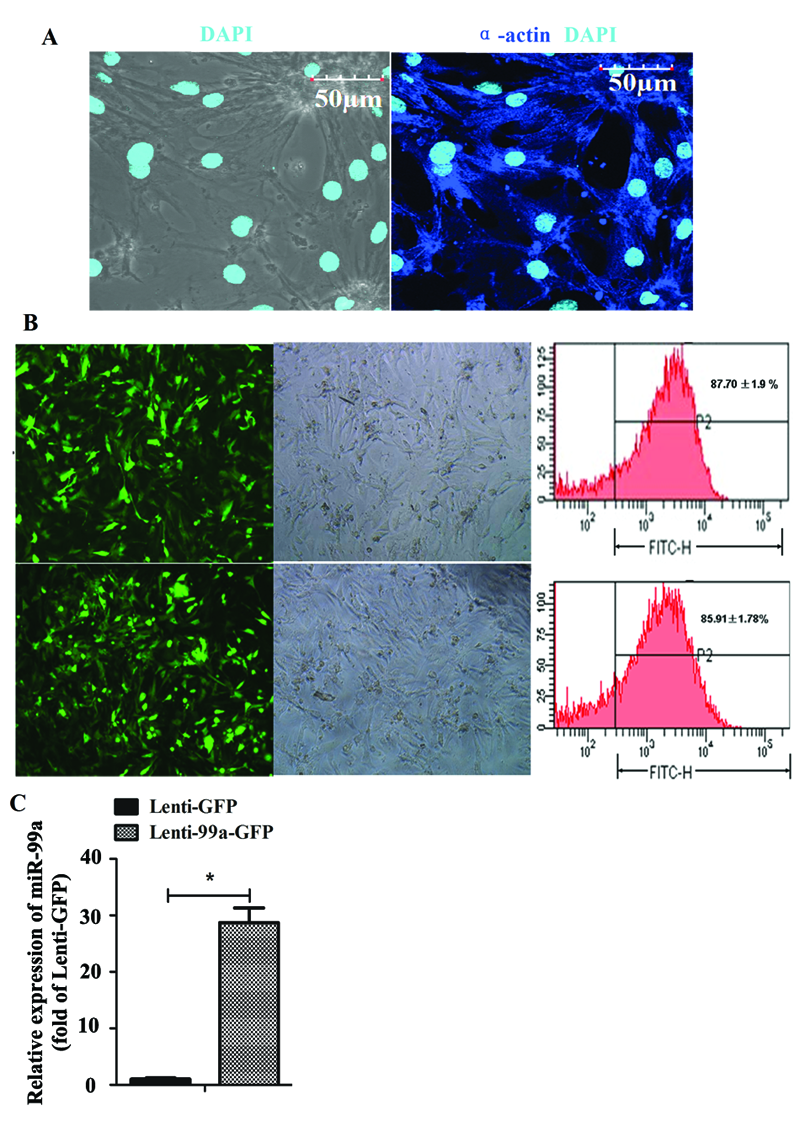

Supplement: Supplementary file 1 — Figure S1 Purity and lentiviral infection of NMVMs. [file jcmm0018-0919-SD1.tif]

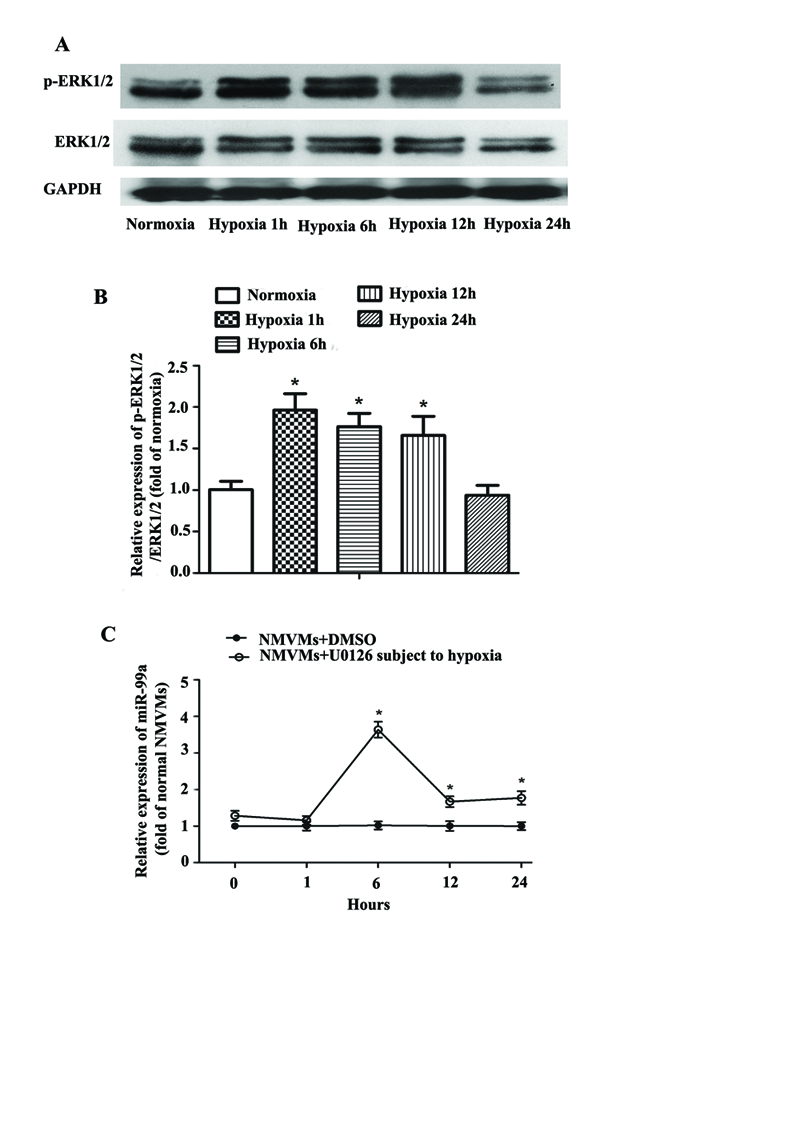

Supplement: Supplementary file 2 — Figure S2 The activity of ERK1/2 was up-regulated under hypoxia. [file jcmm0018-0919-SD2.tif]

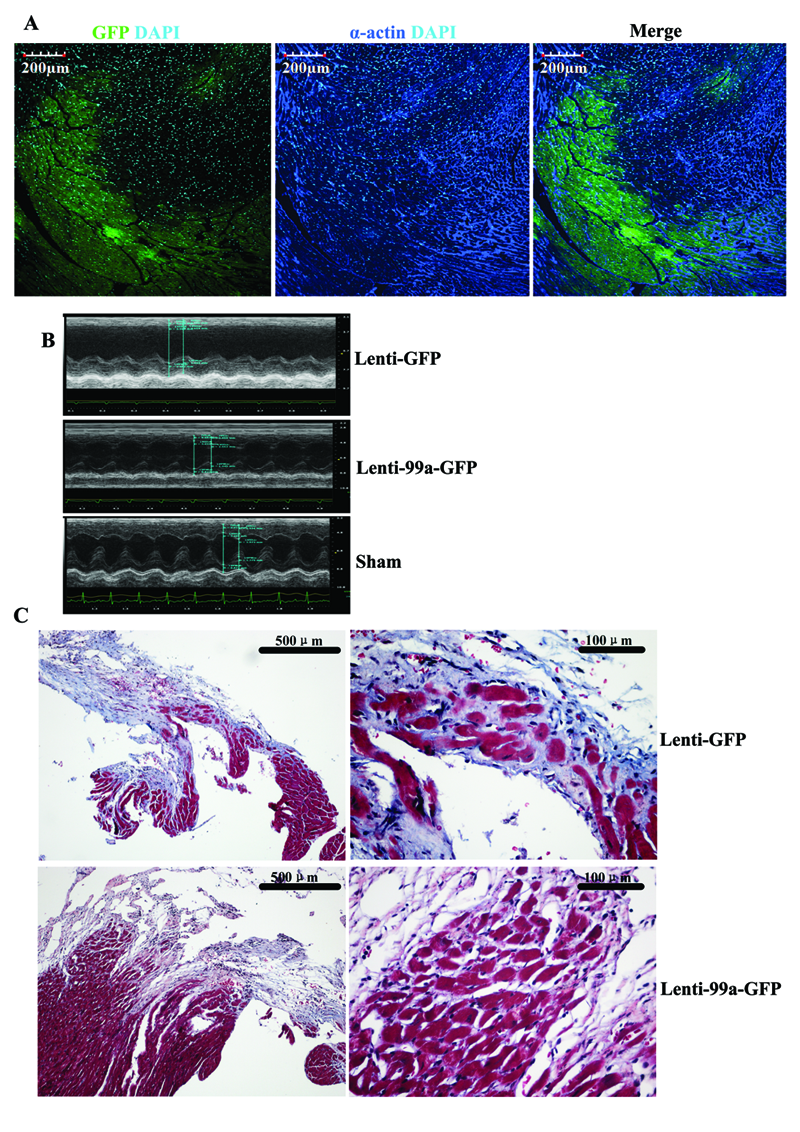

Supplement: Supplementary file 3 — Figure S3 In vivo lentivirus expression. [file jcmm0018-0919-SD3.tif]
