# Supplementary material for: Overexpression of microRNA-99a attenuates heart remodelling and improves cardiac performance after myocardial infarction
Source: J Cell Mol Med. 2014 Mar 13;18(5):919–28. doi: 10.1111/jcmm.12242 (PMC4119397; doi:10.1111/jcmm.12242)
Supplement: Supplementary file 4 — Data S1 Material and methods. [file jcmm0018-0919-SD4.doc]

**Supporting Information**

**1. Material and methods**

**1.1** **Animal Care and Procedure**

The investigation conforms with the Guide for the Care and Use of Laboratory Animals published by the US National Institutes of Health (NIH Publication No. 85–23, revised 1996) and was approved by the Ethics Review Board for Animal Studies of Nanjing Drum Tower Hospital (DTH ERBA 56.01/042D/2011).

**1.2 Recombinant Lentivirus Construction**

Mouse genomic DNA was a gift from Prof. J Li (Nanjing Medical University, China). We designed the primer to get DNA including miR-99a sequence. MiR-99a sequence forward 5'- GAAGACCTTTACTGGGAATA-3', reverse 5'-GCCTTGAATGGCTCTGCTAC-3'. Negative control: TTCTCCGAACGTGTCACGT (Genepharma, Shanghai). Pljm1-egfp plasmid (a gift from Prof. J Li) driven by CMV promoter was connected with miR-99a sequence [1]. This vector also encodes green fluorescence protein (GFP) for the selection of stably infected clones. Successful cloning was confirmed by sequencing. We also accessed the successful overexpression of miR-99a in 293FT cells (Invitrogen) by Taqman RT-PCR. Thereafter, miR-99a sequence was inserted into Pglv3/h1/gfp+puro plasmid vector (Genepharma, Shanghai) and driven by H1 promoter. The pseudoviral particles were produced using lentivector packaging system (Genepharma, Shanghai) according to the manufacturer's instructions. NMVMs were infected at a multiplicity of infection (MOI) of 50.

**1.****3** **Culture of** **Primary NMVMs**

Newborn CD1 mice (1-2 day old) were sacrificed by decapitation. Hearts were quickly excised, and the ventricles were dissected and transferred into ice-cold Hank’s balanced salt solution (HBSS) without Ca^2+^ and Mg^2+^ (Gibco, Invitrogen, Carlsbad, CA) , then rapidly minced in Trypsin-EDTA 0.125% (Invitrogen) at 4°C overnight (within 24 hours) as described [2]. Collagenase (Invitrogen, 0.5 mg/ml in DMEM) was used to further digest the tissues in shaking bath at 37°C within 10 minimums. NMVMs were collected 1.5 hours later from the supernatants and cultured in DMEM containing 1g/L glucose plus 10% FBS and 1% penicillin/streptomycin (GIBCO). The protocol was approved by the Institutional Animal Care and Use Committee of the affiliated Drum Tower Hospital of Nanjing University Medical School.

**1.****4 Cell Infection and Hypoxic Conditions**

Hypoxia was achieved by placing NMVMs in a hypoxia chamber filled with 5% CO _2_, 1% O_2_, and 94% N_2_ at 37°C. NMVMs were cultured for 2 days under normoxia before treatment with hypoxia. NMVMs were harvested at different time points during hypoxic treatment for the analysis of miR-99a levels and the apoptosis. MiR-99a level was evaluated by TaqMan MicroRNA Assay, and cell apoptosis was assessed by Annexin Assay (Biolegend) and DeadEnd Fluorimetric TUNEL System (Roche).

**1.****5 *In Vivo* Study**

To investigate the expression of miR-99a in the border zone of heart after MI, animals were randomly divided into two groups, of which one group received ligation of the left anterior descending coronary artery (LAD) and the other group underwent thoracotomy without LAD ligation (sham). The border zones of infarcted hearts were collected at 1-hour, 3-hour, 1-day, 3-day, 7-day, 28-day and 44-day after surgery (n=5 each time point per group) and the expression of miR-99a in the border zone was determined by Taqman RT-PCR kit (Applied Biosystems, has-miR-99a 000435; U6 001973).

To evaluate the effect of miR-99a treatment on mice with MI, animals were intramyocardially injected with lentivirus containing either miR-99a precursor GFP (lenti-99a-GFP, n=43) or miR-scramble GFP (lenti-GFP, negative control, n=46) in the border zone immediately after LAD ligation [3]. A total of 25µl virus (2.5*10^7^ viral particles) was injected into 3 regions of the border zone using a 29-gauge Hamilton syringe. The sham group (n=29) animals only underwent thoracotomy and pericardiotomy without LAD ligation or lentivirus injection. To explore the effect of MiR-99a on cardiac function under non-surgical conditions, a subset of mice that underwent thoracotomy without LAD ligation (sham) were intramyocardially injected with 25µl of lenti-GFP (n=5) or lenti-99a-GFP (n=6) at 5 sites.

All surgery was performed under aseptic conditions, and mice were anesthetized using ketamine hydrochloride (50 mg/kg) and diazepam (2.5 mg/kg). Mice were sacrificed and hearts were collected at 3-day, 5-day, 7-day and 28-day after MI for Western blotting analysis (n=5 each time point per group). To investigate the long-term expression and therapeutic effect of lentiviral vectors, a subgroup of mice was sacrificed at 90-day after MI (n=3 each time point per group).

Survival of animals in three groups (lenti-99a-GFP, n=25; lenti-GFP, n=28; sham, n=11) was recorded over 28 days after MI. After the 28-day observation period, ultrasonic cardiogram (UCG) was performed on the surviving animals. Hearts were arrested in diastole by intravenous administration of 2mol/l KCl and collected after UCG for further analysis.

**1.6 UCG and Hemodynamic Assessment**

Mice cardiac function was evaluated by transthoracic UCG before, 3 days, 1 week and 4 weeks after viral delivery in a blind fashion. UCG was performed using VisualSonics Vevo2100 ultrasound system equipped with a 30-MHz transducer.

The M-mode measurements of LV dimensions were averaged from more than 3 cycles. LV end-systolic diameter (LVESD) and end-diastolic diameter (LVEDD), interventricular septal thickness in diastole (IVSd) and LV posterior wall thickness (LVPWT) were measured. Percent LV fractional shortening (%FS) was calculated as follows: %FS = (LVEDD-LVESD)/LVEDD*100 (%). Percent ejection fraction (%EF): %EF = 100*((LV Vol;d-LV Vol;s)/LV Vol;d); LV Vol;d = ((7.0/(2.4+LVID;d))*LVID;d^3^); LV Vol;s = ((7.0/(2.4+LVID;s))*LVID;s^3^).

Mice were maintained in a 37 °C metal chamber during the measurement of systolic blood pressure (SBP), diastolic blood pressure (DBP) and heart rate. SBP, DBP and heart rate were measured by a programmable tail-cuff sphygmomanometer (BP-2006A; Softron, Tokyo, Japan). Training measurements were made for 3 days to acclimatize the animals to the machine, followed by 2 days of recorded measurements. Three sets of 5 measurements were taken daily for each mouse, and the 1st set was discarded. To eliminate bias caused by struggling or other physiological alterations, each set of measurements was accepted only if the standard deviation of the set was < 9 mmHg.

**1.****7 MicroRNA and mRNA Quantification**

Total RNA was extracted using TRIzol (Invitrogen) according to the manufacturer’s instructions. For microRNA RT-PCR, TaqMan microRNA assays (Applied Biosystems) were used to quantify the expression of mature miR-99a (Assay ID 000435) and U6 (Assay ID: 001973). MiR-99a expression was relative to the control U6. Amplication and detection were performed using 7500HT Fast Real-Time PCR system (Applied Biosystems). Relative expression was calculated using the comparative Ct method (2^-[△][△]Ct^)[4].

The sequences of primers used for PCR are listed in supplemental Table 1. The reactions contained 2 × SYBR Premix Ex TaqTM (Takara), 50 × Rox Reference Dye II, each primer at 200 nM, and 2μL of cDNA template in a 20μL reaction volume. Amplification was performed with an initial denaturation step at 95°C for 30 s, followed by 40 cycles of denaturation at 95°C for 5 seconds, annealing and extension at 60°C for 34 seconds. After amplification, the amplification specificity was confirmed by melting-curve analysis of the PCR products. The qRT-PCR assay was performed using the ABI Prism 7500 device. All samples were run in triplicate and averaged. GAPDH was chosen as the house keeping gene.

**1.****8 Western Blotting Analysis**

Adult male C57/BL6 mice (8 weeks old) were sacrificed at 3-day, 7-day, 14-day, and 28-day after MI. Then, heart samples were collected. Proteins were extracted from these hearts and assessed by western blotting analysis. The primary antibodies used were antibodies against P70/S6K, FGFR3, GAPDH and β-actin (Bioworld Technology, Inc.), phosphor-P70/S6K and Caspase 3 (Cell Signaling Technology, Inc.), LC3 (Sigma, Inc.), mTOR and ANP (Abcam, Inc), SMARCD1 and SMARCA5 (ProteintechGroup, Inc.).

**1.9 Electron Microscopy**

Hearts were quickly and carefully dissected from MI mice received lentiviral delivery . The border of LV was cut into 1 mm^3^ cubes and fixed with 2% glutaraldehyde in 0.1 mol/L sodium phosphate buffer (pH 7.4) overnight at 4°C. The fixed samples were then post-fixed in 1% OsO_4_, embedded, sectioned and analyzed by transmission electron microscopy.

**1.10 Annexin V Binding Assay**

Annexin V binding assay were performed after 72 hours of infection with lentivirus carrying miR-99a precursor (lenti-99a-GFP) or miR-scramble (lenti-GFP) using a annexin V-APC/PI kit (Biolegend, Inc) according to the manufacturer's instructions. To prepare the cell samples for flow cytometry, cells were gently washed two times with annexin-binding buffer. To each plate, 0.5 ml of Trypsin (0.25%) was added, and the plates were incubated until the cells appear detached by microscopic evaluation. Cells were released from the plate with gentle tapping and added to the collected cells from the medium. Cells were suspended in cold binding buffer and stained with annexin V APC and PI . A total of 10,000 cells was used for analysis of annexin V-APC and PI staining by CellQuest software (Becton-Dickinson, Rutherford, NJ), respectively.

**1.11 TUNEL Assay**

Cellular apoptosis was evaluated by DeadEnd Fluorimetric TUNEL System (Roche) according to the manufacturer’s instructions. Paraffin-embedded tissue sections were counterstained with mouse monoclonal α-sarcomeric actin antibody (1:75, Abcam) and Alexa Fluor 633 goat anti-mouse antibody (1:250, Molecular Probes) as secondary antibody. Cell nuclei were incubated with 4’-6-diamidino-2-phenylindole (DAPI) (Sigma), and then the sections were mounted and analyzed with a Fluoview 1000 confocal microscope (Olympus, Japan). The number of TUNEL-positive cardiomyocytes nuclei was manually determined. The total number of nuclei (exhibited as DAPI-positive signals) was automatically calculated using Image Pro Plus software (Media Cybernetic).

**1.12 Histological Examination**

Hearts were collected 4 weeks after myocardial infarction, fixed with formalin, embedded with paraffin and cut into 4 µm slices for subsequent TUNEL examination, histological and immunofluorescence analysis. The left ventricular diameter was measured in two perpendicular axes and averaged for each animal [5]. LV end-diastolic diameter (LVDd) was measured as described [6]. Myocyte size was measured using cross sections midway between the base and apex of LV with hematoxylin /eosin (HE) staining. Infarct size at day 28 was analyzed as described by Pfeffer *et al* [7], and calculated as percentage of the whole LV with Image J software (ImageJ, NIH, Bethesda, Maryland).

**1.15 Statistical Analysis**

Data were expressed as mean ± SEM. All data analysis was performed using SPSS 16.0 software. Statistical significance was defined as P<0.05 (two-tailed). The normality or otherwise of distribution of the continuous variables was assessed with the Shapiro-Wilk test. Comparison of parameters between two groups was performed by unpaired Student's t test (when distributions were normal) or Mann-Whitney U test (when distributions were significantly skewed). Comparison of mortality between two groups was performed by Kaplan-Meier analysis. Comparison of parameters among three groups was performed by ANOVA or Kruskal-Wallis tests for normally and non-normally distributed data, respectively.

**References**

[1] **Deng LY, Wang JP, Gui ZF, Shen LZ**. Antitumor activity of mutant bacterial cytosine deaminase gene for colon cancer. *World J Gastroenterol*. 2011; 17(24):2958-64.

[2] **Gallina C, Dolgetta S, Alloatti G, *et al.*** Development of morphology and function of neonatal mouse ventricular myocytes cultured on a hyaluronan-based polymer scaffold. *J Cell Biochem*. 2012; 113: 800–7.

[3] **Liang D, Li D, Xin C, *et al*.** Increased expression of integrin-linked kinase attenuates left ventricular remodeling and improves cardiac function after myocardial infarction. *Circulation.* 2009; 120: 764-73.

[4] **Livak KJ, Schmittgen TD.** Analysis of relative gene expression data using real-time quantitative PCR and the 2-^[△][△]^Ct method. *Methods*. 2001; 25: 402–8.

[5]. **Woo YJ, Panlilio CM, Cheng RK, *et al*.** Therapeutic delivery of cyclin A2 induces myocardial regeneration and enhances cardiac function in ischemic heart failure. *Circulation.* 2006; 114: 206-13.

[6] **Fujii T, Nagaya N, Iwase T, Murakami S, *et al.*** Adrenomedullin enhances therapeutic potency of bone marrow transplantation for myocardial infarction in rats. *Am J Physiol Heart Circ Physiol.* 2005; 288: H1444-50.

[7] **Pfeffer MA, Pfeffer JM, Fishbein MC, *et al*.** Myocardial infarct size and ventricular function in rats. *Circ Res.* 1979; 44: 503–12.

Table 1. Gene symbol, name and primer sequences

| Primer | Forward primer 5' to 3' | Reverse primer 5' to 3' |
| --- | --- | --- |
| Atg5 | CCTGAAGATGGAGAGAAGAG | GGACAATGCTAATATGAAGAAAG |
| Atg12 | CCTCGGAACAGTTGTTTATT | CAGGACCAGTTTACCATCAC |
| GAPDH | CGTCCCGTAGACAAAATGGT | TTGATGGCAACAATCTCCAC |

Figure Supplemental I: Purity and lentiviral infection of NMVMs. A. The purity of α-sarcomeric actin + cells was ≥95% in culture cell population. B. NMVMs were infected by lenti-99a-GFP or lenti-GFP. The efficiency of lentiviral infection is 87.70±1.91% in lenti-99a-GFP group and 85.91±1.78 % in lenti-GFP group, as estimated by fluorescence microscopy (left pannel) and flow cytometry (right pannel). C. Expression of miR-99a in NMVMs was assessed by RT-PCR analysis 72 hours after lentivirus infection. MiR-99a expression was 28.76±2.41-fold higher in the lenti-99a-GFP group compared to the lenti-GFP group as control (n=5, * P<0.01).

Figure Supplemental II: A-B. The activity of ERK1/2 was upregulated under hypoxia. B. NMVMs were exposed to 1% oxygen for different time periods. NMVMs were treated with U0126/DMSO for 48 hours, then were subject to hypoxia. MiR-99a expression was increased in U0126 group. *, P<0.05

Figure Supplemental III: A. *In vivo* lentivirus expression. Four weeks after lentivirus delivery in mice heart, we observed immunofluorescence for GFP *in vivo* . B. Representative UCG of mice with LAD ligation in lenti-99a-GFP, lenti-GFP, or sham group 4 weeks after surgery. C. Four weeks after surgery, miR-99a overexpression reduced interstitial fibrosis in the border zone.
